# Bi-Directionality of the Microbiota-Gut-Brain Axis in Patients With Functional Dyspepsia: Relevance of Psychotherapy and Probiotics

**DOI:** 10.3389/fnins.2022.844564

**Published:** 2022-02-28

**Authors:** Sophia Kristina Rupp, Andreas Stengel

**Affiliations:** ^1^Department of Psychosomatic Medicine and Psychotherapy, University Hospital Tübingen, Tübingen, Germany; ^2^Charité Center for Internal Medicine and Dermatology, Department for Psychosomatic Medicine, Charité - Universitätsmedizin Berlin, Corporate Member of Freie Universität Berlin, Humboldt-Universität zu Berlin and Berlin Institute of Health, Berlin, Germany

**Keywords:** functional dyspepsia, gut-brain axis, microbiome, psychotherapy, probiotics

## Abstract

Functional dyspepsia is one of the most commonly diagnosed disorders of the gut-brain interaction worldwide. The precise pathogenesis of functional dyspepsia is complex and remains incompletely understood. Therefore, advances in the understanding of functional dyspepsia could change clinical practice. The aim of this review is to highlight the relevance of psychotherapy and probiotics in the context of the microbiota-gut-brain axis in the pathophysiology and especially in the treatment of functional dyspepsia. Therefore, studies which have been conducted to investigate the role of psychotherapy and probiotics in FD and the microbiota-gut-brain axis in the pathophysiology of functional dyspepsia were examined, and the outcomes of this research summarized. There might be a link between changes in the microbiome and functional dyspepsia. Even though, specific alterations in the microbiome that may be pathognomonic in functional dyspepsia remain unclear, the use of probiotics became a viable treatment option for patients with functional dyspepsia. Since mental illness also plays an important role in the pathophysiology of functional dyspepsia, psychotherapy is a useful treatment method, with additional study results indicating that psychotherapy may also shift the microbiome in a favorable direction. Moreover, other findings suggest that probiotics can be used not only to alleviate gastrointestinal symptoms in functional dyspepsia, but also to treat or even prevent mental disorders in these patients. In summary, in this review we highlight the bi-directionality of the microbiota-gut-brain axis in the pathophysiology of functional dyspepsia. Although there are multiple treatment approaches, the burden of disease in patients with functional dyspepsia is still enormous and a definitive therapy to cure this disease does not (yet) exist. Lastly, there is a lack of studies on the impact of dysbiosis, mental health and probiotics on pathophysiology and symptomatology in functional dyspepsia which should be investigated in future studies.

## Introduction

Functional dyspepsia (FD) is one of the most commonly diagnosed disorders of gut-brain interaction (DGBI) worldwide (Stanghellini et al., 2016) and affects up to 7.2% of people in the community (Sperber et al., 2021). The Rome IV diagnostic provide the basis for defining and classifying patients with DGBI (Schmulson and Drossman, 2017). Here, dyspepsia encompasses various symptoms referable to the gastrointestinal (GI) tract such as postprandial fullness, epigastric pain, or early satiety. In approximately 80% of patients suffering from dyspepsia there is no structural explanation for their symptoms, which is then termed FD (Ford et al., 2015). FD can be further divided into the subgroups epigastric pain syndrome (EPS), characterized by epigastric pain or burning, and postprandial distress syndrome (PDS), characterized by postprandial fullness or early satiety, with overlaps also constituting a subgroup (Stanghellini et al., 2016).

The pathogenesis of FD remains still largely unclarified. However, it is assumed that a disturbed gut-brain axis leads to motility disorders, visceral hypersensitivity, and changes in mucosal and immune function (Vanheel and Farré, 2013; Stanghellini et al., 2016; Jung and Talley, 2018). Moreover, it has now been recognized that FD is closely associated with duodenal eosinophilia, epithelial barrier disruption (Nakagawa et al., 2020) as well as mucosal inflammation accompanied by higher levels of mast cells (Wang et al., 2015) with post-infectious FD possibly representing an own entity (Tack and Talley, 2013; Futagami et al., 2015). As epidemiological studies show, the prevalence of anxiety and depression is higher in patients suffering from FD than in healthy people, indicating that mental disorders may play a role in the development of FD (van Oudenhove and Aziz, 2013). Risk factors suspected so far include female gender, smoking, Helicobacter pylori (HP) infection, acute gastroenteritis, mental disorders, and use of non-steroidal anti-inflammatory drugs (Ford et al., 2015).

Moreover, some patients with DGBI who do not have elevated anxiety scores at baseline show increased anxiety scores later, indicating that the central nervous system and the gut interact in a bidirectional way in functional GI disorders. Moreover, the gut might directly cause brain dysfunction in a subset of these patients (Qin et al., 2014). Furthermore, dysbiosis is a potential factor in the pathophysiology of FD (Ding et al., 2020).

There is little evidence that lifestyle change or physical activity improves symptoms, and although some foods have been linked to the development of symptoms, there is little data on the impact of diet in patients with FD (Duncanson et al., 2018). Drug therapy is therefore the mainstay of treatment but, apart from patients who respond to HP eradication, offers only modest and often temporary relief. Advances in the understanding of FD could change clinical practice, and treatment of microbiome alterations could lead to a cure for a subset of these patients in the future. As the GI microbiome plays a key role in the gut-brain axis, this review discusses the role of the microbiota-gut-brain axis in FD, focusing on possible mechanisms and treatment approaches.

The aim of this narrative review is to highlight the relevance of the microbiome-gut-brain axis in the pathophysiology and especially in the treatment of FD. Many studies have shown that the microbiome is altered in patients with FD and that psychotherapy is also a useful treatment option – we aim to show a link between these two approaches via the microbiome-gut-brain axis and to highlight a potential triad between disordered microbiome, mental illness and FD that could stimulate further research in the future.

## Materials and Methods

Scientific literature on this topic up to October 2021 was collected and screened in the databases Pubmed and PsychInfo using the search terms “functional dyspepsia” or “dyspepsia” combined with “microbiome,” “microbiota,” “dysbiosis,” “bacterial,” “bacterial overgrowth,” “probiotics,” “psychological,” “psychotherapy,” or “cognitive behavioral therapy.” All studies containing material relevant to the topic were considered. There was no date restriction (all studies until October 24th, 2021), but the search was limited to texts in English. After searching the database with the above keywords, 3.122 articles were found. Excluded were review articles, surveys, case reports, commentaries, letters or posters, and studies that deviated from the main topic. Ultimately, 46 articles were selected for this narrative review.

## The Microbiome and Its Role in the Pathophysiology of Functional Dyspepsia

The last decade has witnessed a tremendous increase in research on the trillions of microorganisms living in the human body and their interaction with the host. Since the members of the microbiota are considered essential to the host’s physiological functions, dysregulation can lead to a disruption of microbial-host homeostasis and cause disease (Zmora et al., 2019). Therefore, it is crucial to further elucidate the relevance of the gut microbiota in the development of human disease. The gut microbiota could be a potential target for effective personalized medicine for several diseases in the future. Recent observations have shown that patients with FD show low levels of duodenal and systemic inflammation, especially duodenal eosinophilia (Talley et al., 2007; Liebregts et al., 2011) and increased duodenal permeability (Vanheel et al., 2014), so changes in the microbiota are a factor of interest.

### Alterations in Gastrointestinal Microbiota Associated With Functional Dyspepsia

A previous study compared the gastric fluid composition of patients with FD with healthy controls and reported a significant decrease in the frequency of the genus *Prevotella* in the FD compared to the control group (Nakae et al., 2016). The cause of this dysbiosis could be delayed gastric emptying, which potentially alters the acidity, mucus consistency and partial oxygenation of the stomach, altering the bacterial colonization of the stomach. Furthermore, microbiota of the gastric fluid in patients suffering from FD showed an increased ratio of *Bacteroidetes* to *Proteobacteria*, while no *Acidobacteria* could be detected. However, the gastric fluid of healthy individuals contained *Acidobacteria* and was characterized by a lower ratio of *Bacteroidetes* to *Proteobacteria* (Igarashi et al., 2017). In addition, a greater increase in the proportion of bile acid-positive gastric fluid samples was found in patients with FD than in the control group. Since the reflux of bile acids from the duodenum into the stomach physiologically occurs during gastric motility (Sjövall, 2011), patients with FD may suffer from a disturbed gastric motility. Furthermore, the increased species richness suggests that the mass and diversity of the gastric fluid microbiota is large enough for the metabolites and components of the bacteria to affect the stomach. Thus, it could be suggested that the toxic bacterial cellular components of the gut, such as lipopolysaccharides, stimulate leukocytes to produce pro-inflammatory cytokines, triggering gastric inflammation and consequently increase mucosal permeability, which may lead to gastric (enteric) nervous system dysfunction (Galanos et al., 1985). Since lipopolysaccharides and bile acids increase the permeability of the mucosa, inflammation in the duodenal region in patients with FD could be caused by the refluxed fluid containing such potentially toxic substances (Igarashi et al., 2017).

However, when comparing the microbiome of the upper GI tract in patients with FD and healthy control subjects the levels of *Streptococcus* were higher in the oral cavity, esophagus, stomach, and duodenum of the FD group. Moreover, *Streptococcus* abundance as well as *OTU 90* frequencies correlated positively with upper GI complaints suggesting a link between *Streptococcus* and GI symptoms in patients with FD. In addition, the FD group showed higher levels of phylum *Firmicutes* (Fukui et al., 2020). In line with these findings, in a different study comparing the microbiota of the duodenal mucosa in patients with FD and in healthy subjects, the most prevalent genus in the duodenal mucosa was also *Streptococcus* in both groups. Here, an inverse relationship was noted between the relative frequency of *Streptococcus* and *Prevotella*, *Veillonella* and *Actinomyces*, which were significantly lower in patients with FD. In addition, quality of life and total bacterial load were also evaluated in patients with FD. Here, a negative correlation was shown between the bacterial load of the duodenal mucosa and quality of life. Furthermore, as the bacterial load on the mucosa increased, so did the severity of symptoms after a standardized meal and bacterial diversity declined with increasing total bacterial count (Zhong et al., 2017). Aforementioned studies illustrate that microbial alterations in FD are not confined to one site in the GI tract, highlighting the potential importance of homeostatic imbalance in the pathogenesis of these diseases.

Moreover, an investigation of the composition of the microbiome by analyzing fecal samples of rats with FD and liver depression-spleen deficiency syndrome, showed elevated levels of *Firmicutes*, *Proteobacteria* and *Cyanobacteria* in this model, while a lower frequency of *Bacteroidetes* was seen in the model compared to the control group (Qiu et al., 2017). When interpreting these results, it should be noted that the model may not exactly represent a model for FD and rather mimics chronic stress. Therefore, cautious interpretation of these data is warranted.

In another study, small intestinal bacterial overgrowth (SIBO) – an overload in the number and/or type of colonic bacteria in the upper GI tract (Bures et al., 2010; Sachdev and Pimentel, 2013) – was detected in 2 out of 38 (5.3%) patients with DGBI (one subject suffered from FD, the other from an overlap of FD and irritable bowel syndrome [IBS]) using the glucose breath test (Shimura et al., 2016). IBS is also a DGBI and is diagnosed according to the Rome IV criteria based on recurrent abdominal pain associated with defecation or in association with an altered stool frequency or form (Mearin et al., 2016). However, regarding the usefulness of the hydrogen breath test in diagnosis of FD, it should be noted that an H_2_ increase can also occur in patients without GI symptoms or complaints.

In contrast, a recent meta-analysis including seven studies showed that the prevalence of SIBO is significantly higher in patients with FD compared to healthy individuals. The studies used breath tests (glucose breath test and lactulose breath test) to detect SIBO, and overall, the prevalence of SIBO in patients with FD was 32.7%. The prevalence of SIBO within the different subgroups of FD (EPS, PDS, overlap of EPS and PDS) was also investigated - no significant differences were identified here (Gurusamy et al., 2021). A case-control study showed that the prevalence of SIBO in patients with FD was 14%, compared to 10% in healthy controls. An analysis of the different FD subgroups did not take place here. Although the results of this study were not statistically significant, the researchers concluded that SIBO may cause more discomfort in patients suffering from FD than in healthy individuals due to increased visceral sensitivity (Chuah et al., 2021). Similarly, a recent study in patients with FD using quantitative cultures of the proximal small intestine to detect SIBO showed a higher overall prevalence of SIBO in patients with FD than in healthy subjects. Moreover, analysis of the different subtypes of FD showed a prevalence of SIBO of 12.5% in EPS, 20.8% in PDS and 31.6% in EPS-PDS overlap (Tziatzios et al., 2021). The results of these studies indicate that SIBO may be associated with the development or exacerbation of symptoms in a subgroup of patients with FD. Whether SIBO is the causal factor or a consequence of another process, e.g., impaired mucosal permeability, requires further research. However, it may be important to consider SIBO as a differential diagnosis in the examination of patients with refractory GI symptoms in routine clinical care. Even though these results do not provide a specific analysis of the composition of the microbiome, the findings do point toward a potential significance of GI dysbiosis in the pathophysiology of FD.

In addition, in a small subgroup (5%) of patients with FD, infection with HP appears to be the cause of dyspepsia (Moayyedi et al., 2000; Ding et al., 2020). Since HP infection often leads to chronic mucosal inflammation of the stomach and duodenum, disturbances in GI motility and sensitivity often follow (Suzuki and Moayyedi, 2013). Considering that dysregulation of the gut microbiota and gut-brain axis may play a crucial role in the pathogenesis of FD, the basis of microbiota-gut-brain axis dysfunction in the treatment of FD cannot be neglected.

In contrast, a recent study showed that the microbial composition of patients with FD was similar to those of healthy controls, covering a largely overlapping spectrum (Vasapolli et al., 2021). Thus, there appears to be limited consensus on the existence of FD-associated microbiome signatures. This may be due to the different methodologies used in assessing the microbiome, different sampling sites and 16S rRNA sequencing compared to shotgun metagenomics. While the use of 16S profiles of the microbiota is biased due to the selection of primers targeting specific variable regions of the 16S rRNA gene, shotgun metagenome sequencing assures that all DNA signatures are captured, in addition to allowing prediction of the functional capacity of the microbiota (Teh et al., 2021). All studies mentioned here utilized 16S rRNA sequencing with different primer targets, which limits the comparability of the results (Table 1).

**TABLE 1:** Methods used for assessing the microbiome and outcomes of the studies discussed in this review (in alphabetical order).

First author	Method	Main findings in patients with functional dyspepsia compared to healthy subjects
Fukui et al., 2020	16S-rRNA sequencing, Primer: 341F, 805R	*Firmicutes* ↑, *Streptococcus* ↑
Igarashi et al., 2017	16S-rRNA sequencing, Primer: 341F, 806R	*Acidobacteria* ↓, *Bacteroidetes* > *Proteobacteria* ↑
Nakae et al., 2016	16S-rRNA sequencing, Primer: 516F, 1510R	*Bifidobacterium* ↑, *Prevotella* ↓
Qiu et al., 2017	16S-rRNA sequencing, Primer: 515F, 806R	*Bacteroidetes* ↓, *Cyanobacteria* ↑, *Firmicutes* ↑, *Proteobacteria* ↑
Vasapolli et al., 2021	16S-rRNA sequencing, Primer: not indicated	no differences
Zhong et al., 2017	16S-rRNA sequencing, Primer: 917F, 1392R	*Actinomyces* ↓, *Prevotella* ↓, *Streptococcus* ↑, *Veillonella* ↓

*↑, increased; ↓, decreased.*

When interpreting all these aforementioned alterations in the microbiome composition, it should always be kept in mind that there is no evidence of a causal relationship between dysbiosis, and FD and that further research is needed. Even though the studies discussed above show several changes in the microbiota composition and suggest a link, it should be taken into account that a microbiome shift can also be caused by pharmacotherapeutic treatment, e.g., proton pump inhibitors (PPIs) (Perry et al., 2020) or antidepressants (McGovern et al., 2019) for pain management.

### Probiotics as a Promising Treatment Option?

If we assume dysbiosis as a cause of FD symptoms, probiotics might be considered as a possible treatment. Therefore, several studies have been conducted to shed further light on this possible option: One study included 131 participants suffering from HP-associated FD who were randomly assigned to receive *Lactobacillus gasseri OLL2716* (LG21)-containing yogurt or placebo yogurt once daily for 12 weeks. Although the primary endpoint of this randomized double-blind trial, namely a decrease in HP load, was not significant, the results showed that postprandial fullness was significantly reduced in the LG21 group at the end of the study compared to baseline and compared to the placebo group (Takagi et al., 2016). Similar results were found in another randomized double-blind controlled study, where HP uninfected participants consumed yogurt containing LG21 for 12 weeks. Here, the LG21 group showed a statistically non-significant improvement in gastric symptoms compared to the control group, suggesting that this probiotic may be a useful therapeutic treatment for patients with FD (Ohtsu et al., 2017). However, no improvements were found in EPS-like symptoms by LG21. Instead, the results suggest that LG21 has a stronger positive effect on PDS symptoms than on EPS-like symptoms. Therefore, it can be assumed that prokinetic agents may have a positive effect on PDS (Matsueda et al., 2010), and the positive effects of LG21 may focus mainly on delayed gastric emptying and impaired adaptability to relaxation. Even though the effects of yogurt on gut microbiota are still controversial (Elli et al., 2006), the improvement in upper abdominal symptoms could be due to a reduction in secretory bile acids in the duodenum (Beeckmans et al., 2020). More precisely, hydrophobic bile salts affect the integrity of the small intestine and increase intestinal permeability (Erickson and Epsten, 1988; Stenman et al., 2013), which can consequently lead to bacterial translocation (Banerjee et al., 2016; Bednarska et al., 2017). A recent study found a positive correlation between the ratio of secondary to primary bile salts and duodenal permeability in patients with FD (Beeckmans et al., 2020). These results underline that the effects of certain bile salts on duodenal permeability and their potential role in the pathophysiology of FD should be further investigated in the future.

However, not only the symptom severity decreases, but also the microbiome shows a transformation with LG21 administration: In patients with FD, the dysbiosis in the gastric fluid was resolved by consuming yogurt containing LG21. Interestingly, an increase in the frequency of *Prevotella* was associated with a decrease in the severity of PDS symptoms in patients with FD receiving LG21 yogurt. This observation leads to the hypothesis that the frequency of the genus *Prevotella* could be used as a biomarker for the outcome of FD treatment (Nakae et al., 2016). In line with these findings, a different study reported that probiotic therapy with yogurt containing LG21 in patients with FD resulted in a change in the composition of the gastric fluid microbiota toward that observed in healthy subjects (Igarashi et al., 2017).

A different study that administered *Lactobacillus paracasei L-37* in form of a drink for 14 and 28 days showed that this administration reduces the severity of symptoms in patients with FD while significantly alleviating clinical symptoms such as abdominal pain and belching. In addition, the number of probiotic bacteria such as *Lactobacillus, Lactococcus* and *Weissella* increased, while the number of pathogenic bacteria such as *Lachnoclostridium* significantly diminished (Sun et al., 2021). Interestingly, this study also measured gut metabolites. Here it was shown that consumption of *Lactobacillus paracasei L-37* was associated with an increase in beneficial metabolites such as pelargonic acid, benzoic acid, and short-chain fatty acids (SCFAs), while harmful gut metabolites such as hippuric acid decreased (Sun et al., 2021).

Another randomized double-blind, placebo-controlled study investigated the effect of *Bacillus coagulans MY01* and *Bacillus subtilis MY02* administration in FD patients. The primary endpoint, a decrease in the score of the Leuven Postprandial Distress Scale, was achieved in the intervention group. Interestingly, while this study did not find a pronounced change in microbiome composition, it did show that an increase in *Faecalibacterium* could be associated with probiotic efficacy. In addition, a reduction in SIBO was shown in the intervention group, which can be inferred from the reduced proportion of positive glycolic acid breath tests in patients with FD taking PPIs (Wauters et al., 2021). Although studies are still scarce, the findings described above showing that probiotics have a positive effect on the severity of FD symptoms could imply a link between dysbiosis as a cause and FD symptoms as a consequence.

Moreover, there are several studies investigating the efficacy of treating FD with traditional Chinese medicine, which also point to a role of the microbiome in the pathophysiology of FD (He et al., 2019; Zhang et al., 2019, 2020; Gao et al., 2020). These studies should be interpreted with caution when it comes to the transferability of the results to humans with FD. The findings are not discussed in detail because the animal models used here likely do not reflect the complex pathophysiology of FD.

Now it remains unclear which specific mechanisms are behind the potentially positive effects of probiotics on FD symptomatology. Probiotics are defined by The Food Agricultural Organization/World Health Organization as “Live microorganisms which, when administered in adequate amounts, confer a health benefit on the host.” It is speculated that probiotics inhibit pathogenic bacteria in the gut, decrease epithelial permeability, decrease visceral hypersensitivity, improve gut motility, and alter the mucosal stress response, thereby reducing symptom severity in GI disorders (Hosseini et al., 2012). Another hypothesis is that probiotics act in the upper GI tract by reducing the levels of *Escherichia/Shigella*, a main source of toxic lipopolysaccharides, leading to restoration of alterations in the gastric microbiome (Igarashi et al., 2017). In addition, probiotics appear to reduce visceral hypersensitivity by regulating pain receptor expression in the GI tract (Rousseaux et al., 2007). Another trial suggests that a reduction in glycogen synthesis and associated blood lipid lowering is induced by *Lactobacillus paracasei*, and consequently leads to an improvement in gut motility (Gudiña et al., 2010). It is known that increased permeability of the duodenal mucosa plays a role in the pathophysiology of FD (Vanheel et al., 2014). Recently, probiotics have been reported to reduce this increased permeability by ameliorating abnormalities directly in gut microbiota or by producing SCFAs via fermentation (Dalile et al., 2019).

Although the exact mechanisms mediating the effects of probiotics are still largely unknown, SCFAs are considered as possible mediators. SCFAs are metabolites of bacterial fermentation of dietary fibers in the gut and whose neuroactive properties may play an important role in communication along the microbiota-gut-brain axis (Stilling et al., 2016). As mentioned above, probiotics may lead to higher levels of SCFAs via the proliferation of beneficial SCFA-producing bacteria or the fermentation of complex carbohydrates. SCFAs can influence gut-brain communication and brain function directly or indirectly through immunological, endocrine, vagal and other pathways (Dalile et al., 2019). In addition, SCFAs affect systemic inflammation by regulating the secretion of interleukins and interact with vagal afferents (Lal et al., 2001; Corrêa-Oliveira et al., 2016). Data on studies investigating these direct effects in humans are still scarce. However, we know that SCFAs exert a range of effects to improve gut health, such as maintaining intestinal barrier integrity (Daly and Shirazi-Beechey, 2006; Lewis et al., 2010), protection against intestinal inflammation (Lewis et al., 2010) and increase of mucin secretion (Barcelo et al., 2000). Furthermore, SCFAs relax the proximal stomach in humans (Ropert et al., 1996) and additionally influence intestinal motility through the activation of SCFA receptors (Dass et al., 2007), the release of the intestinal hormone peptide YY (PYY) (Cherbut et al., 1998) or SCFA-induced serotonin release from enterochromaffin cells (Fukumoto et al., 2003). Nevertheless, the exact mechanism of action of individual probiotics in FD treatment can only be hypothesized so far.

Taken together, although data are still sparse, there might be a link between changes in the microbiome and FD. Even though specific alterations in the microbiome that may be pathognomonic in FD remain unclear and require further research, the efficacy of probiotics, which are naturally designed to alter the microbiome and reverse dysbiosis, became a viable treatment option for patients with FD. Thus, it appears that dysbiosis may play a considerable role in the pathophysiology of FD.

## Top-Down: Impact of Psychotherapy on FD – a New Perspective

A study of the Swedish population showed that anxiety at baseline increases the risk of developing FD by almost 8 times after 10 years follow-up (Aro et al., 2015). Interestingly, multiple studies highlighted that the prevalence of anxiety and depression is significantly increased in patients with FD compared to healthy people (Talley et al., 1986). These observations indicate that mental illness plays a significant role in the pathogenesis of FD. Furthermore, pathophysiological research indicates that psychosocial factors and mental disorders may play a role in FD by modulating both visceral signal processing in the brain (van Oudenhove and Aziz, 2013; Browning and Travagli, 2014) and the effects of stress hormones on pain perception (Hannibal and Bishop, 2014). Furthermore, it is known that psychosocial factors and stress hormones also affect other aspects of the GI tract such as motility (Venkova et al., 2010), immune system activation (Konturek et al., 2011), permeability (Gareau et al., 2008) and microbiota (Tannock and Savage, 1974; Bailey and Coe, 1999). On the other hand, FD symptoms are thought to induce anxiety or depression due to a cytokine response in low-grade intestinal inflammation, which plays an important role in the development of psychological distress in patients with FD (Koloski et al., 2012). Therefore, psychological therapies have recently been adapted for the treatment of disturbed brain-gut interactions such as FD. Although we will primarily discuss the link between mental health and FD in the following, it is important to note that a prospective study from 2016 showed that two-thirds of patients with IBS or FD who did not suffer from anxiety or depression at baseline showed elevated anxiety and depression scores at 1-year follow-up (Koloski et al., 2016). These results underline that gut-to-brain alterations are relevant in a subgroup of these patients.

A meta-analysis of 2021 investigated the potential beneficial effects of various psychological therapies on the global symptom severity in patients with FD (Rodrigues et al., 2021). Even though the sample sizes in the studies included were mostly small, the effectiveness of psychological therapies on global FD symptom scores was clearly demonstrated (Rodrigues et al., 2021). Most studies included investigated the effect of cognitive behavioral therapy (CBT) on FD symptoms (Wilhelmsen et al., 1994; Cheng et al., 2007; Dehghanizade et al., 2015; Orive et al., 2015; Xiong et al., 2019) and concluded that CBT leads to a significant improvement in the severity of symptoms (Wilhelmsen et al., 1994; Cheng et al., 2007; Orive et al., 2015; Xiong et al., 2019), pain intensity (Orive et al., 2015) and further lowers the impact of the disease on patients’ lives (Dehghanizade et al., 2015). Moreover, a significant increase in gastric emptying rate and alterations in gastric motility parameters were monitored in the intervention group compared to the control group (Xiong et al., 2019). Based on these results, CBT can be considered as an effective therapeutic option in patients suffering from FD. Psychoanalysis also shows positive effects on patients with FD (Hamilton et al., 2000; Faramarzi et al., 2013, 2015) by reducing both FD-associated symptoms rated by patients (Hamilton et al., 2000; Faramarzi et al., 2013, 2015) and treating gastroenterologists (Hamilton et al., 2000). Other psychotherapeutic methods, such as hypnotherapy, are also effective in relieving symptoms (Calvert et al., 2002; Chiarioni et al., 2006) and improving quality of life (Calvert et al., 2002) in patients with FD. Moreover, gut-oriented hypnosis has been shown efficient in shortening gastric emptying time in both dyspeptic and healthy subjects, while patients with FD additionally reported a reduction in symptoms of epigastric fullness and abdominal discomfort (Chiarioni et al., 2006).

We have now highlighted two different factors that might play a role in the pathogenesis of FD – one is dysbiosis, and the other is disordered mental health. Now, one might wonder where the positive effects of psychological therapies in FD come from. On the one hand, as already mentioned above (Koloski et al., 2016), a subgroup of patients with FD first develops a mental disorder in the course of the disease, on which psychotherapeutic approaches can have a positive effect. Secondly, by combining both approaches, dysbiosis and disturbed mental health, one could hypothesize that psychotherapy alleviates mental disorders by shifting the microbiome in a favorable direction. This approach has already been explored in studies, although not specifically in FD models: Here, one study investigated the effect of mindful awareness practice in elderly patients with mild cognitive impairment and found that improvement in the patients’ cognition is associated with alterations in fecal microbiome profile. These results imply that signals from the brain may directly or indirectly modulate the composition of the gut microbiome (Khine et al., 2020). Similarly, interesting results were obtained from an investigation of outdoor nature-related activities and their effects on the intestinal microbiota of preschool children. The children’s gut microbiota changed, especially in terms of *Roseburia* frequency and fecal serotonin levels (Sobko et al., 2020). In contrast, a 12-week aerobic exercise intervention in adolescents with subthreshold mood syndromes and healthy adolescents showed no significant positive effects on gut microbiota in either group. Precisely, there were no differences in intestinal microbiota diversity, genus, or frequencies detected, which could also be due to a potentially inadequate exercise intensity (Wang et al., 2021). In line with these results, the combination of three months of dietary education combined with CBT also showed no changes in the diversity or composition of the gut microbiome compared to usual care in patients with IBS (Kamp et al., 2021).

An important aspect that should be mentioned in the context of mental condition on dysbiosis and GI discomfort is the role of the vagus nerve. It is known that stress increases gut permeability and alters the composition of the GI microbiome via various neuromodulators (Taché and Bonaz, 2007; Tache et al., 2018), a mechanism that also plays a role in the pathophysiology of IBS. A study in rats has shown that chronic stress alters intestinal permeability, which consequently leads to dysbiosis and later to visceral hypersensitivity (Moussaoui et al., 2017). Interestingly, stress can reduce vagus nerve activity (Taché and Bonaz, 2007; Wood and Woods, 2007), which may promote GI inflammation (Bonaz et al., 2016). Although there are no data yet on the effect of vagal stimulation on the GI microbiome, it can be speculated that the vagus nerve may have an impact on the gut microbiome through its effects on intestinal permeability (Meregnani et al., 2011; Karl et al., 2017). The efferents of the vagus nerve may have an anti-inflammatory effect in the gut and at the same time reduce intestinal permeability – both effects may be attributed to a strengthening of the tight junctions through vagal activity. Study results imply that vagus nerve activity exerts a protective function on the intestinal epithelial barrier (Cheadle et al., 2014; Yu and Li, 2014), but the exact mechanism is still unclear. Conversely, this means that reduced vagal activity increases epithelial permeability and consequently promotes systemic inflammation and chronic disease. Stress could therefore abolish the protective effect of the vagus nerve on the epithelial barrier as a whole and thus promote dysbiosis by disrupting epithelial homeostasis (McEwen, 2008). Furthermore, a reduction in vagal tone has been demonstrated in IBS and inflammatory bowel disease (Pellissier et al., 2014). The effects of psychotherapy on the microbiome and GI symptoms in FD could therefore also be explained by the influence of the vagus nerve. Consequently, monitoring and targeting vagal tone in patients with DGBI could be of value to potentially restore a homeostatic microbiota-gut-brain axis (Bonaz et al., 2018).

Now one can wonder whether the prevalence of psychological morbidity varies within the different subgroups of FD. Even before the Rome III subdivision of FD into subgroups, it was shown that mood and anxiety disorders were more common in patients with non-pain-dominant FD than in patients with pain-dominant FD (Handa et al., 1999). Another study, however, showed a correlation between epigastric pain and neuroticism, abuse, and somatization (Fischler et al., 2003). In contrast, other studies observed a relationship between PDS and somatization, anxiety, and depression, which was not observed in EPS (Aro et al., 2009; Hsu et al., 2009). Moreover, a study from 2012 showed that depression was only associated with PDS (Clauwaert et al., 2012). These findings highlight the urgent need for future studies focusing on a possible link between psychological comorbidity and specific symptoms in patients with FD, so that treatment approaches for FD in the future will hopefully be more personalized and based not only on symptoms but also on underlying pathophysiology and -psychology.

In conclusion, a precise answer to the hypothesis that psychotherapy alleviates mental disorders by shifting the microbiome in a favorable direction cannot be given based on the current data. The studies mentioned here included only small sample sizes, and the results are poorly comparable due to different patient characteristics and different interventions. To further confirm or reject this hypothesis, there is an urgent need for studies that further investigate these interesting effects of psychotherapy on the microbiome specifically in patients with FD, so that we are able to better understand this direction of the brain-gut-microbiome axis in the future.

## Bottom-Up: the Microbiome and Its Role in Mental Health

We are aware that patients with FD often suffer from mental illness (Talley et al., 1986) and that anxiety can increase the risk for FD (Aro et al., 2015). The increased prevalence of anxiety and depression in patients with FD could be due to the disease burden or the FD symptoms themselves, which could trigger anxiety or depression through a cytokine reaction resulting in low-grade intestinal inflammation.

A growing body of evidence suggests that the gut microbiota communicates with the central nervous system, possibly through neural (vagus nerve, spinal cord), endocrine (HPA axis), metabolic (SCFAs, bile acids, tryptophan and many more), and immunological pathways (cytokines), thereby influencing brain function (Cryan and Dinan, 2012). Furthermore, microbiota release neuroactive compounds, such as GABA, serotonin, dopamine, and acetylcholine, thereby acting locally on the enteric nervous system (Lyte, 2011; Sarkar et al., 2016). Some of these neuroactive substances access the brain through the blood and the circumventricular organs or via the vagus nerve. Therefore, it could be hypothesized that a disturbed microbiome might affect mental health, followed by anxiety and depression. Thus, the mental disorders might be a consequence of the dysbiosis and therefore promote the development of FD, which may explain the findings that anxiety increases the risk of FD (Aro et al., 2015) and observations indicating that psychosocial factors and mental disorders may play a role in FD by modulating visceral signal processing in the brain (van Oudenhove and Aziz, 2013; Browning and Travagli, 2014).

Although the following section focuses on the impact of the microbiome on mental health and the associated potential of probiotics and fecal microbiota transplantation (FMT) in the treatment of FD, it should be noted that this axis also works in the other direction: Psychosocial stress is known to affect the GI tract at multiple sites. Psychosocial stress not only affects the microbiome (Tannock and Savage, 1974; Bailey and Coe, 1999), but also other aspects of the GI tract, such as motility (Venkova et al., 2010), immune system activation (Konturek et al., 2011), and permeability [74.].

In a study on patients with IBS 65% of the participants suffered from increased psychological distress, 31% from anxiety, and 21% suffered from depression. Interestingly, microbial composition was significantly associated with distress and depression suggesting a link between certain taxa and mental condition (Peter et al., 2018). Furthermore, while depression was negatively associated with *Lachnospiraceae* frequency, participants who scored higher on distress, anxiety, depression, and stress perception had significantly higher abundances of *Proteobacteria*. In addition, patients with anxiety showed elevated levels of *Bacteroidaceae* (Peter et al., 2018). The microbiome signature observed in this study in psychologically distressed patients with IBS could also exist for patients with FD. The in-depth characterization of a microbiome signature specific to psychologically distressed patients with FD could lead to the discovery of new biomarkers and therapeutics. Future studies are needed to further investigate this theory. However, in such future studies, it should be kept in mind that the precise microbial signature of FD has not yet been fully elucidated, so the results must be interpreted with caution: Here, it might be challenging to distinguish whether a potential microbial alteration is associated with mental disorders or with FD, which could consequently lead to the signature of mental disorders being falsely attributed to FD. It is therefore of great relevance not only to demonstrate an FD-specific microbial signature, but also to demonstrate a clear link between microbial alterations and both specific FD symptoms and established pathophysiological manifestations.

## Probiotics and Their Impact on Mental Health

Several studies show that probiotics have positive effects on cognitive abilities (Allen et al., 2016; Roman et al., 2018; Lew et al., 2019; Berding et al., 2021; Bloemendaal et al., 2021) and mood (Allen et al., 2016; Roman et al., 2018; Lew et al., 2019; Berding et al., 2021), therefore they were termed ‘psychobiotics’. Moreover, probiotics have also been shown to alter brain activity in magnetoencephalography (Wang et al., 2019).

There are also studies, although not specifically in the context of FD, that underline how probiotics influence anxiety, depression, and other mental disorders: In depressed patients, administration of a probiotic containing *Lactobacillus helveticus* and *Bifidobacterium longum* leads to an improvement in depression scores compared to the placebo group (Kazemi et al., 2019). In addition, administration of the same probiotic formula to healthy volunteers for thirty days resulted in relief of the subjects’ psychological distress associated with decreased cortisol levels (Messaoudi et al., 2011). Furthermore, administration of a probiotic containing *Lactobacillus plantarum PS128* (PS128) to a group of patients with self-reported insomnia resulted in significant improvement of depression scores, fatigue, brainwave activity and awakening during deep sleep (Ho et al., 2021). Consistent with these findings, a study on sixty Japanese medical students taking *Lactobacillus gasseri CP2305*-containing tablets or placebo tablets observed a significant decrease in anxiety and sleep disturbances compared to the placebo group (Nishida et al., 2019). Similar results were found in a group of stressed adults where the administration of *Lactobacillus plantarum P8* led to a decrease in anxiety accompanied by a reduction of pro-inflammatory cytokines such as interferon (IFN)-γ and tumor necrosis factor (TNF)-α (Lew et al., 2019). Another randomized double-blind study conducted on 423 women investigated the effect of *Lactobacillus rhamnosus HN001* (HN001) on postnatal mood. Here, taking HN001 resulted in lower depression and anxiety scores, suggesting that this probiotic may be effective in treating postnatal depression and anxiety (Slykerman et al., 2017). In contrast, one study investigated the effect of a probiotic containing *Bifidobacterium bifidum W23, Bifidobacterium lactis W51, Bifidobacterium lactis W52, Lactobacillus acidophilus W37, Lactobacillus brevis W63, Lactobacillus casei W56, Lactobacillus salivarius W24, Lactococcus lactis W19* and *Lactococcus lactis W58* compared to placebo in depressed patients and could not find a difference regarding depressive symptoms between groups (Chahwan et al., 2019).

### Fecal Microbiota Transplantation and Its Impact on Mental Health

Another frequently discussed approach, instead of probiotic therapy, is fecal stool transfer to restore the balance of the gut microbiota: To address this hypothesis, a study was conducted to investigate the effect of FMT from healthy patients to patients with IBS. Interestingly, four weeks after FMT, patients with IBS showed a significant increase in the diversity of their microbiota, while their psychological status also improved. The researchers concluded that FMT is a potential method to restore the balance of the gut microbiota in patients with IBS (Mizuno et al., 2017). This finding should be confirmed in larger follow up randomized controlled studies. These results also raise the question of whether FMT could be a promising treatment for FD-associated dysbiosis and, at the same time, therapy or even prevention of common mental disorders in patients with FD. To our knowledge, there are no FD-related data to date that have investigated this interesting approach.

Where do the beneficial effects of probiotics (and possibly also FMT) on mental disorders come from? There are several mechanisms by which the gut microbiome influences brain function (Mayer et al., 2015). For example, it has been shown that gut bacteria can alter systemic levels of neurotransmitter precursors and thus influence central neurotransmitters (Desbonnet et al., 2010). It has also been shown that *Candida, Streptococcus* and *Enterococcus* can produce neurotransmitters such as serotonin (Lyte, 2013, 2014; Wall et al., 2014; Yano et al., 2015), *Bacillus* and *Saccharomyces* species can produce noradrenaline (Dinan and Cryan, 2017), while *Lactobacillus* and *Bifidobacterium* species can synthesize and release GABA (Dinan and Cryan, 2017). These microbially synthesized neurotransmitters can act locally and also cross the intestinal mucosa to act locally but potentially also the central nervous system via nerval signaling.

The positive effects of probiotics and FMT on anxiety and depression may be explained by the competitive exclusion of harmful gut pathogens, the decrease in proinflammatory cytokines and communication with the central nervous system via vagal sensory fibers, leading to changes in neurotransmitter levels or function (Yan and Polk, 2002; Lammers et al., 2003; Ramiah et al., 2008; Forsythe et al., 2010). Interestingly, *Lactobacillus helveticus R0052* was shown to protect the microflora of the digestive tract from invasion by pathogenic bacteria (Wine et al., 2009). It is also known that *Clostridium* and *Bacteroides spp*. produce propionic acid, a SCFA that increases aggression and fear in animals (Hanstock et al., 2004). In addition, *Lactobacillus plantarum* has been shown to suppress intestinal pathogens, increase the population of beneficial gut microbiota, accompanied by increased levels of SCFAs in adults (Wang et al., 2014; Kwok et al., 2015).

As mentioned earlier, SCFAs are thought to play a major role in the interplay with the gut-brain axis. However, SCFAs do not only act on the gut, but also in psychopathological processes, which could explain the link between the microbiome and mental health. For example, fecal SCFA levels have been shown to be lower in depressed patients than in controls (Skonieczna-Żydecka et al., 2018; Szczesniak et al., 2016), while another study reported that fecal butyrate correlated with reported emotional problems in children (Michels et al., 2017). Furthermore, sodium butyrate has been shown to reverse depression-like and mania-like behaviors in rats (Resende et al., 2013). We do know that SCFAs can directly affect the brain, as they can cross the blood-brain barrier (Mitchell et al., 2011). Although their uptake into the brain appears to be minimal, they can centrally modulate neurotrophic factor levels (Schroeder et al., 2007), modulate neurotransmission (Frost et al., 2014), and promote serotonin biosynthesis (Fukumoto et al., 2003; Yano et al., 2015). Taken together, the interaction of SCFAs with these gut-brain pathways may directly or indirectly modulate processes associated with neuronal function, learning, memory, and mood (Intlekofer et al., 2013; Varela et al., 2015). To date, studies investigating the direct effect of SCFAs on brain function have been limited to *in vitro* and animal studies, making transferability to humans difficult. In the future, it should be further clarified whether the SCFAs derived from the microbiota can reach physiologically relevant concentrations in the human CNS at all and whether these SCFAs (derived from the microbiota) can also trigger these effects.

Moreover, the role of inflammatory processes in emotion is supported by evidence of a link between depression and elevated levels of Interleukin (IL)-6, TNF and C-reactive protein (Alesci et al., 2005). In addition, antidepressants are thought to work in part through the production of the immunoregulatory cytokine IL-10, thus suppressing inflammation and depressive mood (Maes, 2001). Interestingly, *Lactobacillus* and *Bifidobacterium* stems attenuated inflammatory responses or led to IL-10 production in rodents (Desbonnet et al., 2008; Duncker et al., 2008; Karimi et al., 2009) and showed anti-inflammatory activities in human cell lines (Wallace et al., 2003). In addition, *Bifidobacterium* administration has been shown to suppress an increase in plasma tryptophan, which has been associated with depression in animal models (Desbonnet et al., 2008). While the role of cytokines in FD or their association with symptom severity is still unclear, a significant increase in TNF-α and IL-1β was observed in one study (Liebregts et al., 2011). Moreover, while there was no difference in IL-6 levels between patients with FD and healthy subjects (Kindt et al., 2009; Liebregts et al., 2011), IL-6 levels were associated with increased abdominal pain (Kindt et al., 2009). Interestingly, it was found that patients with higher duodenal cytokine levels are more likely to benefit from therapeutic interventions targeting gut dysbiosis, particularly probiotics (Leventogiannis et al., 2019). However, these findings need to be further elucidated in future studies.

In addition, a previous study on *Lactobacillus plantarum PS128* (PS128) showed that taking PS128 can increase dopamine and serotonin levels in the brains of mice and both neurotransmitters are affected by common antidepressants (Markou et al., 1998). Moreover, normal hypothalamus-pituitary-adrenal (HPA) axis activity is regulated by circadian excitatory inputs, stress-induced stimulation and several negative feedback loops mediated by corticotrophin-releasing factor (CRF), adrenocorticotropic hormone and cortisol (Munck et al., 1984). In one of the studies discussed here, daily administration of probiotics resulted in a significant reduction in urinary free cortisol levels (Messaoudi et al., 2011). Although the mechanism underlying the stress-reducing effect of *Lactobacillus gasseri CP2305* is unclear (Nishida et al., 2019), the strain is known to colonize the gut, and it has been reported that administration of heat-inactivated cells to the stomach or intestine of rats can activate the afferent vagus nerve (Nishida et al., 2019). Thus, these properties may stimulate the gut-brain axis directly or indirectly and alter the activity of the HPA axis, leading to lower stress-related symptoms and an improvement of the gut environment.

To further explore the underlying mechanism of how probiotics affect brain physiology and thus anxiety and depression, animal models were employed: Here, changes in the expression of GABA receptors in the brains of mice were demonstrated together with changes in anxiety-related behavior upon treatment with *Lactobacillus rhamnosus* (Bravo et al., 2011), and it was suggested that the vagus nerve might be the link between the gut and altered GABA receptor expression in the brain. Other studies examining similar effects in humans showed no differences in modifying stress-related measures underlining the difficulty of extrapolating from animal to human studies (Kelly et al., 2017).

In summary, these results are difficult to compare due to the use of differing questionnaires, heterogeneous patient groups and the different composition of probiotics. Nevertheless, the few IBS-related data give hope that probiotics are also a treatment option in patients with FD. The results mentioned above raise the idea that probiotics can be used not only to alleviate GI symptoms in FD, but also to treat or even prevent mental disorders in these patients. So far, there is a lack of FD-specific studies on this topic.

## Conclusion

**FIGURE 1:**
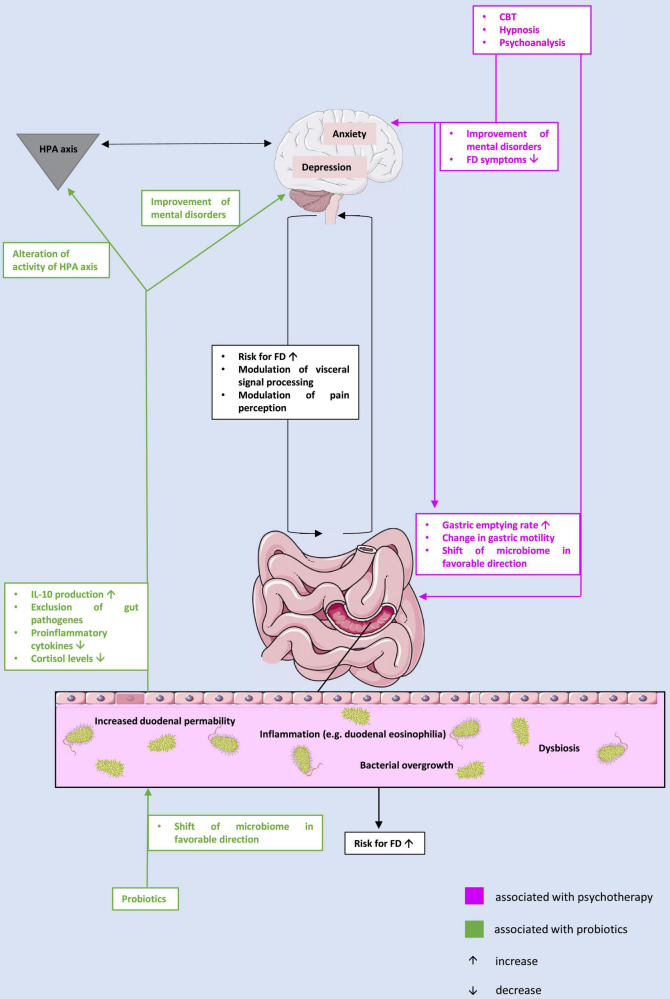
The potential interplay between microbiome, gut, and brain in the context of the pathophysiology of functional dyspepsia and treatment approaches. CBT, cognitive behavioral therapy; FD, functional dyspepsia; HPA, hypothalamus-pituitary-adrenal; IL, interleukin.

In summary, in this review we have highlighted the bi-directionality of the microbiota-gut-brain axis in the pathophysiology of FD: First, we demonstrated that there may be a link between changes in the microbiome and FD. Although the specific changes in the microbiome that may be pathognomonic in FD remain unclear and require further research, the efficacy of probiotics, which are inherently designed to alter the microbiome and reverse dysbiosis, is a viable treatment option for patients with FD. Thus, it appears that dysbiosis plays a potentially key role in the pathophysiology of FD (Figure 1).

On the other hand, we have illuminated that mental illness both plays a role in the pathogenesis of FD and can occur for the first time in the majority of patients during the course of FD, which is why psychological therapies are also a promising approach in the treatment of FD symptoms. One could link both approaches, dysbiosis and mental disorders, and hypothesize that psychotherapy shifts the microbiome in a favorable direction. This latter approach has already been explored in studies, although not specifically in FD models. However, a precise answer to this hypothesis cannot be given based on the current data. To further confirm or reject this hypothesis, studies are urgently needed to further investigate these interesting effects of psychotherapy on the microbiome specifically in patients with FD, so that we can better understand this direction of the brain-gut-microbiome axis in the future.

There is growing evidence that the gut microbiota communicates with the central nervous system, possibly through neural, endocrine, and immunological pathways, and influences brain function. Therefore, it could be hypothesized that a disrupted microbiome could impact mental health, followed by anxiety and depression. Thus, the mental disorders could be a consequence of the dysbiosis and thus favor the development of FD, which could explain the findings that anxiety increases the risk of FD. The findings mentioned above raise the idea that probiotics can be used not only to alleviate GI symptoms in FD, but also to treat or even prevent mental disorders in these patients.

In summary, the complex pathophysiology of FD remains still largely unexplained. Although there are multiple treatment approaches, the burden of disease in patients with FD is still enormous and a definitive therapy to cure this disease does not yet exist. There is a lack of studies on the impact of dysbiosis, mental health and probiotics on pathophysiology and symptomatology in FD. Therefore, well-designed studies are needed in the future, to elucidate more precisely the underlying causes of dysbiosis, mental health disorders and FD or, where appropriate, to explore a potential direct link between them. Resolving one of these factors could also give rise to a possible link between one or more pathophysiological factors and FD symptomatology.

## Author Contributions

SR performed the database search, screened the papers, and wrote the first draft of the manuscript. AS planned the article and gave critical input throughout the study. Both authors finalized the manuscript.

## Conflict of Interest

The authors declare that the research was conducted in the absence of any commercial or financial relationships that could be construed as a potential conflict of interest. The reviewer AM declared a past collaboration with one of the author AS to the handling editor.

## Publisher’s Note

All claims expressed in this article are solely those of the authors and do not necessarily represent those of their affiliated organizations, or those of the publisher, the editors and the reviewers. Any product that may be evaluated in this article, or claim that may be made by its manufacturer, is not guaranteed or endorsed by the publisher.
